# Relationship Between Total 25-Hydroxyvitamin D and Parathyroid Hormone Concentrations During Early Gestation in Indian Women

**DOI:** 10.3390/nu17162626

**Published:** 2025-08-14

**Authors:** Nandini Chopra, Fathima Ayoob, Roopashree C, Shashikala Karanth, Manjula Harish, Annamma Thomas, Vasista Adiga, Annapurna Vyakarnam, Catherine Hawrylowicz, Anura V. Kurpad, Pratibha Dwarkanath

**Affiliations:** 1 Centre of Doctoral Studies, Manipal Academy of Higher Education, Manipal 576104, India; nandini.c@sjri.res.in; 2 Division of Nutrition, St. John’s Research Institute, St. John’s National Academy of Health Sciences (a Unit of CBCI Society for Medical Education), Bengaluru 560034, India; roopac@sjri.res.in; 3 Division of Epidemiology & Biostatistics, St. John’s Research Institute, St. John’s National Academy of Health Sciences (a Unit of CBCI Society for Medical Education), Bengaluru 560034, India; fathima.a@sjri.res.in; 4 Department of Gynaecology & Obstetrics, St. John’s Medical College Hospital, St. John’s National Academy of Health Sciences (a Unit of CBCI Society for Medical Education), Bengaluru 560034, India; shashikala.k@stjohns.in (S.K.); manjulaharish68@gmail.com (M.H.); rejiann@gmail.com (A.T.); 5 Division of Infectious Diseases, St. John’s Research Institute, St. John’s National Academy of Health Sciences (a Unit of CBCI Society for Medical Education), Bengaluru 560034, India; vasista.k@sjri.res.in (V.A.); annapurna.v@sjri.res.in (A.V.); 6 Peter Gorer Department of Immunobiology, School of Immunology & Microbial Sciences, King’s College London, London SE1 7EH, UK; catherine.hawrylowicz@kcl.ac.uk; 7 Department of Physiology, St. John’s Medical College, Bengaluru 560034, India; a.kurpad@sjri.res.in

**Keywords:** vitamin D, pregnancy, parathyroid hormone, breakpoint

## Abstract

**Background:** A high prevalence of vitamin D deficiency (VDD) during early pregnancy has been reported globally, along with a high risk of adverse pregnancy and birth outcomes. The present cut-off to diagnose VDD during pregnancy is <20 ng/mL of serum 25-hydroxyvitamin-D (25(OH)D) concentration, but there is a lack of consensus on this value. We evaluated this diagnostic cut-off specifically during early pregnancy among apparently healthy Indian women. **Methods:** Demographic details, obstetrics history, anthropometric measurements, and blood samples were collected from 395 apparently healthy pregnant Indian women at ≤14 weeks of gestation, after obtaining written informed consent. The inverse relationship between 25(OH)D and parathyroid hormone (PTH) concentrations was examined to define the breakpoint at which PTH was maximally suppressed using a segmented regression analysis. Covariate exposures associated with VDD were also examined. **Results:** The breakpoint at which a sharp increase in PTH was observed in response to decreasing 25(OH)D concentrations occurred at 15.76 ng/mL (95%CI: 12.3–19.2; *p* < 0.001). Using this diagnostic threshold, 66.1% of pregnant women were VDD compared to 82.0% when using the present cut-off. Statistically significant associations between VDD and parity (*p* = 0.011), season (winter: *p* = 0.001; post-monsoon: *p* < 0.001), anemia status (*p* = 0.044), and physical activity (*p* = 0.045) were also found. **Conclusions**: Our diagnostic cut-off for VDD, derived from PTH regulation in early pregnancy, is lower than the currently recommended threshold. Although assessing vitamin D status may be challenging due to the influence of modifiable and non-modifiable factors such as parity, anemia, season, and physical activity. These findings underscore the need to re-evaluate existing cut-offs through well-designed longitudinal studies to prove causality between this threshold and adverse pregnancy outcomes.

## 1. Introduction

The global burden of vitamin D deficiency (VDD) varies substantially from 15.7 to 76.6% based on geography [1]. In India, despite abundant sunlight, the prevalence of vitamin D insufficiency (20–30 ng/mL) and deficiency (<20 ng/mL), assessed as serum 25-hydroxyvitamin-D (25(OH)D), remains paradoxically high [2,3,4], with notable geographical variability ranging from 9.4 to 38.8% [5].

While VDD prevalence is influenced by several factors such as ethnicity, skin pigmentation, pollution, and physical activity level [6,7], it is also dependent on the diagnostic cut-off of serum 25(OH)D used. Due to the lack of consensus on the serum 25(OH)D diagnostic cut-off for maintaining calcium homeostasis [8], the relationship between 25(OH)D and parathyroid hormone (PTH) is widely evaluated. Low serum 25(OH)D concentrations are associated with compensatory secondary hyperparathyroidism [9], to maintain calcium homeostasis by mobilizing calcium from bone to serum [10,11]. This inverse relationship therefore tightly regulates blood calcium levels by stimulating renal synthesis of 1,25-dihydroxyvitamin D to increase intestinal calcium absorption through the action of PTH [12]. However, sustained elevation of serum PTH also increases bone calcium resorption, thereby potentially compromising bone health [10,11]. Thus, the point at which serum PTH begins to increase in response to a decline in serum 25(OH)D concentrations has been used to define a diagnostic cut-off for VDD [7,13,14,15]. However, a substantial variation in the identification of inflection points in the 25(OH)D-PTH relation has been noted in previous studies [7,14,16]. This warrants exploration of this relationship in different populations, among whom pregnant women represent a particularly vulnerable group due to their increased physiological demand.

During pregnancy, 25(OH)D not only supports the physiological needs of the mother but also plays a crucial role in fetal skeletal growth and development. VDD in pregnant women has been associated with adverse maternal outcomes, such as, pre-eclampsia, gestational diabetes mellitus, and pre-term birth [17,18], and is also linked to adverse neonatal outcomes, including hypocalcemia and nutritional rickets in the offspring [17,19]. However, factors like parity, anemia, seasonal variation, physical activity, and sun exposure [20,21,22,23] can influence maternal circulating 25(OH)D concentrations. Therefore, defining an inflection point in the relationship between PTH and 25(OH)D during early gestation, while accounting for these covariates, may help establish a diagnostic cut-off for VDD in pregnant Indian women, a population requiring timely and adequate antenatal care [24].

Currently, estimates of VDD prevalence among Indian pregnant women rely on community-based, small studies which have used cut-offs identified in Western general populations as the reference [25,26,27]. Given the variability in the VDD diagnostic cut-off, it is worth establishing if population-specific cut-offs are needed. Therefore, this study aimed first, to identify the breakpoint in the relationship between serum 25(OH)D and PTH concentrations to diagnose VDD in the first trimester of pregnancy in Indian pregnant women, and second, to evaluate associations between vitamin D status and its modifiable and non-modifiable factors.

## 2. Materials and Methods

### 2.1. Study Participants

The present analysis was conducted on 395 apparently healthy pregnant women (18–40 years) who were participants in a parent study funded by Medical Research Council—Global Challenge Research Fund (MRC-GCRF) (Clinical Trials Registry—India (CTRI/2022/01/039091). The trial received ethical approval from the Institutional Ethical Committee of St. John’s Medical College prior to commencement (Ref. No.: IEC/30/2021 dated 3 March 2021).

Participants with ≤14 weeks of gestation at the time of attending the routine antenatal clinic at out-patient unit of the Obstetrics Department of St. John’s Medical College Hospital (SJMCH), Bengaluru, India, were screened for eligibility. Those who gave written consent and agreed to comply with the study protocol, blood collection, and supplement regime (in the parent trial) were included in the present analysis. Pregnant women with multiple/twin pregnancies were excluded. Further, women with any chronic morbidity (like hypertension, diabetes, metabolic syndrome, and thyroid disorder), on medications/treatment, with known infections (such as, HIV, syphilis, HBsAg), assisted conception, on vitamin D supplementation before study commencement, and/or anticipating to discontinue/move out of the study site before the closure, were excluded.

### 2.2. Study Measurements

Information regarding maternal age, education, parity, socioeconomic status, occupation, anthropometry, dietary intake, blood pressure, physical activity, exposure to sunlight, clinical history, ultra-sound scan information, delivery details, birth outcome, and other relevant information was collected throughout the gestational period through interview-based questionnaires.

The basic anthropometric measurements of weight (using the digital calibrated and validated weighing scale to the nearest 10 g, height (using portable stadiometer calibrated to the nearest 0.1 cm), mid upper arm circumference (MUAC) (using the non-elastic measuring tape calibrated to the nearest 0.1 cm,) skinfold thicknesses of biceps, triceps, subscapular and suprailiac (by calibrated Holtain callipers to the nearest 0.1 mm) were taken by a trained research staff. The body composition parameters such as fat percent, fat mass, and fat-free mass were computed using standard formula [28].

A validated physical activity questionnaire was administered once at each trimester to understand the physical activity level (PAL) of the study participants during pregnancy [29]. A questionnaire was adapted to evaluate sun exposure. However, baseline data on sun exposure could not be collected for all participants as it was added after the initial phase of participant recruitment as an amendment to the protocol. Finally, data on duration of direct sun exposure, use of sunscreen, and length of clothing was obtained only for 163 participants.

### 2.3. Laboratory Measurements

Hemoglobin (Hb) levels were measured in whole blood collected in ethylenediaminetetraacetic acid (EDTA)-containing tubes using an automated hematology analyzer (Horiba Medical, Kyoto, Japan). The remaining blood was centrifuged at 3000 rpm for 10 min to separate the plasma. The serum was also separated from the plain Vacutainer using the same method. Both serum and plasma were then aliquoted into pre-labeled cryovials and stored at –80 °C until analysis. The total 25(OH)D and PTH levels in serum samples were measured by the principle of electrochemiluminescence immunoassay (Cobas 6000, Roche Diagnostics, Rotkreuz, Switzerland). The PTH assay used in this study is traceable to the NIBSC 95/646 (WHO) international standard, with mean recovery of 100% ± 4% across the measuring range (40–4000 pg/mL) on Cobas analyzers. Instrument-specific quality controls were employed to ensure the accuracy of the measurements, with an intra- and inter-day coefficient of variation (CV) less than 5% across all levels.

### 2.4. Sample Size and Statistical Analyses

This analysis is part of a parent study that aimed to assess the impact of vitamin D_3_ supplementation on maternal 25(OH)D levels at third trimester and in cord blood; therefore, the sample size for this analysis was participants, who consented to the first trimester screening blood draw and study protocol of the main trial (details mentioned in the Section 2.1). Descriptive statistics for study participants and 25(OH)D levels across seasons are presented as mean (standard deviation) and median (interquartile range) (IQR) for continuous variables. Categorical variables are reported as frequencies and percentages. To assess seasonal differences in 25OHD levels, a one-way ANOVA or Kruskal–Wallis rank sum test was performed, depending on the normality of the data. Pairwise comparisons were conducted using post hoc tests to identify significant differences between seasons.

To determine a breakpoint in the relationship of vitamin D (25(OH)D) and PTH, segmented regression analysis was performed [30]. The statistical significance of the breakpoint was evaluated using Davies’ test [31,32]. Further, univariate logistic regression was conducted to identify the covariates associated with 25(OH)D levels below the breakpoint cut-off. Additionally, univariate linear regression analyses were performed to assess the associations of PTH levels and 25(OH)D concentrations with the same set of potential risk factors. The variables included in both models were age, education, gestational age (according to last menstrual period), parity, body mass index (BMI), weight, body composition, hemoglobin (Hb) concentrations, anemia status, physical activity level, season of blood collection, and dietary calcium and vitamin D intake.

## 3. Results

### 3.1. General Characteristics of Study Population

**Figure 1:**
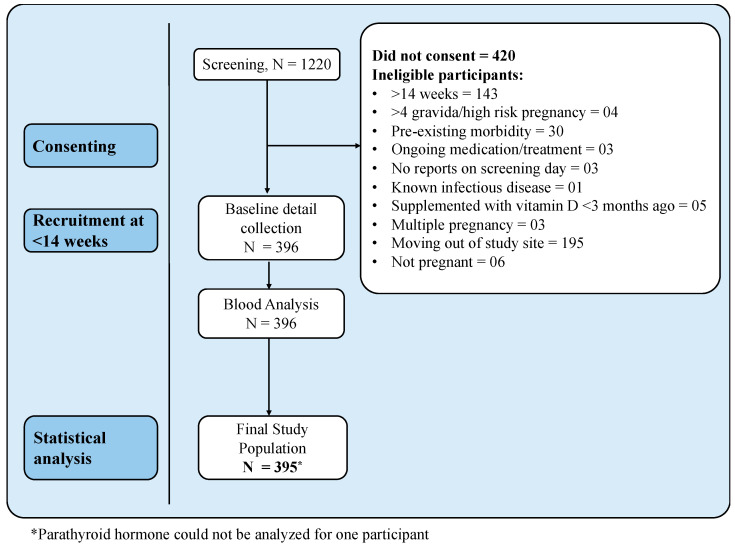
Flow diagram of study participants.

We screened 1220 pregnant women visiting the out-patient department of the obstetrics department of St. John’s Medical College Hospital (Figure 1). Out of these, 396 pregnant women gave informed written consent for participating in the study. However, the final breakpoint analysis was performed on 395 participants as PTH value was missing for one participant. The general characteristics of the study population are given in Table 1. At the time of blood collection, the mean age of participants was 26.3 ± 12.4 years, at 11.6 ± 2.3 weeks of gestation. Sixty-three percent of the pregnant women were nulliparous. The average systolic and diastolic blood pressure was 98.5 ± 10.1 mmHg and 67.9 ± 8.0 mmHg, respectively. The majority (62.6%) of the participants in the study were graduates or post-graduates and 67.7% reported living in nuclear families. All participants reported being non-smokers. None of the participants reported consuming any vitamin D supplement prior to study commencement. Table 1 also provides the anthropometric details of the study participants. The average weight, height, and body mass index (BMI) of the study participants were 57.5 ± 11 kg, 155 ± 5.4 cm, and 23.7 ± 4.2 kg/m^2^, respectively. Average intakes of energy, protein, carbohydrate, and fat were 1861 ± 471 kcal/d, 57.4 ± 16.3 g/d, 285.5 ± 75.4 g/d, and 54.3 ± 17.6 g/d, respectively, while those of iron and calcium were 14.8 ± 4.9 mg/d, and 894 ± 5 mg/d, respectively. Additionally, to account for the dietary vitamin D_2_ habitually consumed through fortified oils by Indians, we estimated its amount based on the brand of oil, level of fortification and the average per day consumption of total oil (Table 1).

The biochemical profile of study participants at recruitment is presented in Table 2. Mean hemoglobin concentration of participants was 11.8 ± 1.3 g/dL. Based on the World Health Organization’s definition of iron deficiency anemia in the first trimester [33], only three participants were severely anemic. The average concentrations of 25(OH)D and PTH were 14.6 ± 8.7 ng/mL and 24.7 ± 12.4 pg/mL, respectively. Concentrations of these biomarkers based on the season of blood collection are provided in Supplementary Figure S1.

### 3.2. Breakpoint Analysis of 25-Hydroxyvitamin D and Parathyroid Hormone

**Figure 2:**
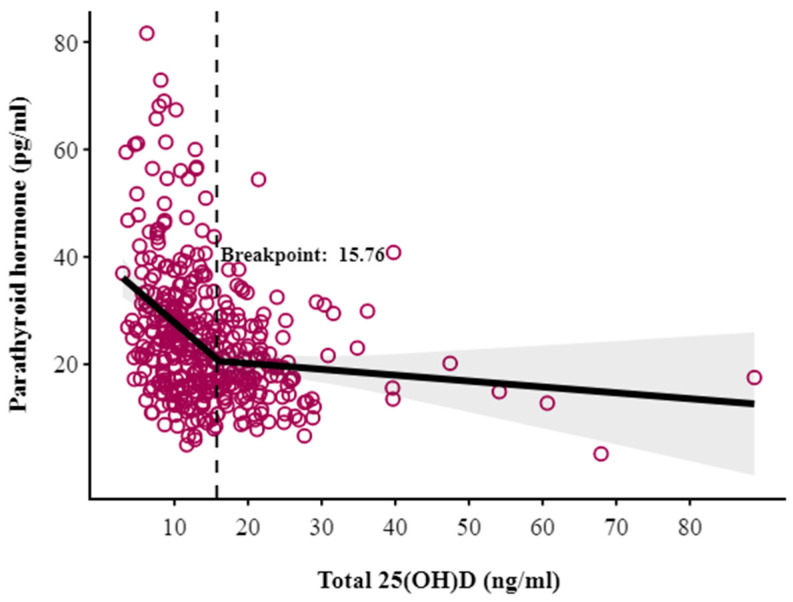
Breakpoint analysis of total 25(OH)D versus parathyroid hormone.

An inverse correlation existed between 25(OH)D and PTH concentrations (Spearman’s r = −0.358, *p* < 0.001). To determine the threshold for VDD, we identified the point at which PTH concentration exhibited a rapid increase. This threshold, referred to as a breakpoint, was determined using segmented regression analysis, at 15.76 ng/mL (95% CI: 12.3 to 19.2; *p* < 0.001) (Figure 2), indicating that below this level, PTH concentrations increased steeply in response to declining 25(OH)D levels. Further, a non-linear, inverse relationship between the PTH:25(OH)D ratio and 25(OH)D concentrations was found, supporting the absence of a hemodilution effect in our study population (Supplementary Figure S2).

### 3.3. Associations with 25(OH)D Status and Parathyroid Hormone Concentration

Table 3 depicts the associations of different exposures with serum 25(OH)D status (greater or less than 15.76 ng/mL) through logistic regression. The odds of having 25(OH)D concentrations that were lower than the cut-off was significantly higher during the post-monsoon (Odds Ratio (OR) = 3.00, 95% Confidence Intervals (CI): 1.621–5.55, *p* < 0.001) and winter seasons (OR = 2.43, 95% CI: 1.41–4.19, *p* = 0.001), compared to the monsoon season. Linear regression analysis also confirmed these findings (Supplementary Table S1). Nulliparous women had higher odds of deficiency compared to multiparous women in early gestation (OR = 1.74, 95% CI: 1.14–2.67, *p* = 0.011). Moreover, compared to women with normal hemoglobin concentration, those with moderate/severe anemia had significantly higher odds of VDD (OR = 2.76, 95% CI: 1.03–7.41, *p* = 0.044). Women with 15–30 min of direct sunlight exposure had significantly lower odds of vitamin D deficiency compared to those with less than 15 min (OR = 0.453, 95% CI: 0.23–0.88, *p* = 0.019). Since the details of this variable could be obtained only for a subset of participants, results were not reported in the table.

The covariates associated with serum PTH concentrations in early gestation were also assessed. PTH was found to be inversely associated with gestational age, decreasing by 0.8 units for each one-unit increase (β = −0.792, 95% CI: −1.313 to −0.271, *p* = 0.003). Levels of PTH were also higher in primiparous women compared to nulliparous (β = 3.218, 95% CI: 0.578 to 5.858, *p* = 0.017). Additionally, each one-unit increase in body mass index (BMI) was associated with a 0.312-unit increase in PTH (β = 0.312, 95% CI: 0.022 to 0.601, *p* = 0.035). PTH levels were also inversely associated with gestational age, decreasing by 0.792 units for each one-unit increase (β = −0.792, 95% CI: −1.313 to −0.271, *p* = 0.003). Additionally, the linear regression analysis revealed a significant positive association between fat-free mass (FFM) and PTH levels, with each one-unit increase in FFM associated with a 0.2 unit increase in PTH (β = 0.204, 95% CI: 0.006 to 0.402, *p* = 0.043). A similar trend was observed for average maternal weight (β = 0.107, 95% CI: −0.004 to 0.218, *p* = 0.059) (Supplementary Table S1).

## 4. Discussion

We conducted a breakpoint analysis to assess the response of serum PTH to declining 25(OH)D concentrations, to determine the inflection point at which PTH began to rise in non-supplemented Indian women in their first trimester of pregnancy (11.6 ± 2.3 weeks). A high variability in this inflection point has been observed in previous studies conducted in different populations and gestational timepoints. For example, Hysaj et al. conducted a study in 203 pregnant women residing in Switzerland and found an inflection point of 25(OH)D at 18.9 ng/mL in the late third trimester [16], while Kazemian et al. employed a one-term fractional polynomial analysis model in 227 pregnant Iranian women and reported a 25(OH)D threshold of 12.48 ng/mL at a median gestational age of 39 weeks [14]. In contrast, Kramer et al. reported a higher inflection point at 32.9 ng/mL (82 nmol/L) at 29.7 ± 2.9 weeks of gestation in a cohort of 468 calcium/vitamin D-supplemented pregnant women [15]. However, most studies were conducted in late pregnancy which observes maximum plasma volume expansion. This, in turn, impacts the concentrations of biomarkers of interest [34]. Moreover, inter-study variations are attributable to population-specific determinants—such as ethnicity, skin pigmentation, latitude, seasonal sunlight availability, cultural clothing practices, air pollution, and use of sunscreen [6,7,35,36].

In our cohort, segmented regression analysis in early pregnancy revealed an inflection point of 15.76 ng/mL of 25(OH)D. Nevertheless, the majority of our participants presented low 25(OH)D levels with normal PTH concentrations, indicating an early stage of VDD. This can be partially explained by the increased renal production of 1,25-dihydroxyvitamin D which helps in maintaining active vitamin D levels, thereby ensuring adequate calcium absorption to preserve calcium homeostasis, without elevating PTH [37]. Also, adequate dietary calcium intake, as observed in our study population (mean intake: 894.5 ± 319.4 mg/day), may have further contributed to the maintenance of normal PTH levels.

Furthermore, since participants were assessed during early gestation, our estimate is not confounded by supplementation or hemodilution (Supplementary Figure S2). Given that plasma volume expansion is most pronounced in the second trimester [34], and its effect on PTH and 25(OH)D is significant only in late pregnancy [38], our study provides a valid basis for examining this relationship in early pregnancy and serves as a reasonably sensitive indicator of VDD burden.

Applying this threshold (<15.76 ng/mL), 66.1% of participants were classified as VDD, compared to the more substantial 82.0% and 96.9% as per the Endocrine Society Clinical Practice Guideline’s threshold of <20 ng/mL and <30 ng/mL [39], respectively. High prevalence of VDD in our population can be attributed to inadequate direct sun exposure, high skin pigmentation, and the cultural practice of wearing covering clothes [35].

In logistic linear regression, VDD status (25(OH)D <15.76 ng/mL) was significantly associated with season during blood collection, parity, and anemia. Participants assessed in summer and monsoon exhibited comparable 25(OH)D levels, whereas those assessed during post-monsoon/winter seasons demonstrated higher odds of VDD when compared to monsoon-recruited participants. This aligns with studies attributing seasonal variations in vitamin D status to fluctuations in exposure to ultraviolet B (UVB) radiation [20].

The relationship between VDD and parity remains inconsistent in literature. While some have reported nulliparous women being at a higher risk of low vitamin D status compared to multiparous women [20,21,40,41], others have observed the opposite [23,42]. In our study, nulliparous women were at an increased risk of VDD than multiparous women. This could be attributed to the dietary or behavioral differences between nulliparous and multiparous women which were not captured in our dataset.

Our study also revealed that moderately/severely anemic participants were at a higher risk of VDD than non-anemic women. Ahmed et al. in a cohort of 515 pregnant women residing in rural Bangladesh reported similar findings [21]. Iron is hypothesized to affect vitamin D metabolism through its involvement in cofactors of several heme-containing monooxygenases, which affect vitamin D levels [43,44]. Previous studies have noted an inverse relationship between serum iron and bone biomarkers [45]. Our study did not find any association between hemoglobin, a marker of iron deficiency, and 25(OH)D concentration (β = 0.09; 95% CI: −0.585 to 0.765; *p* = 0.793; Supplementary Table S1).

Finally, physical activity emerged as a protective factor against VDD, consistent with existing literature [40]. This reflects the indirect impact of physical activity on the cutaneous synthesis of 25(OH)D by exposing individuals to more sunlight [46].

To ascertain the robustness of our estimated breakpoint, we also conducted sensitivity analyses in subsets of data which showed the breakpoint estimate to remain consistent and statistically significant against the variations in the range of 25(OH)D and PTH. A recent study by Rostami et al. supported the cut-off of ≥15 ng/mL of 25(OH)D as protective against adverse pregnancy outcomes [47]. Similarly, Merewood et al. found women with vitamin D levels ≥37.5 nmol/L (15 ng/mL) to be less likely to undergo cesarean section compared to those with lower levels (adjusted Odds Ratio (AOR): 3.84; 95% CI: 1.71 to 8.62) [48]. Contrarily, a systematic review and meta-analysis observed a reduction in risk of adverse outcomes only at 25(OH)D levels of more than 20 ng/mL (*I*^2^  >  50%) [49].

This study has some limitations. Although, a breakpoint in the relationship of serum 25(OH)D and PTH concentrations was identified, our study was not powered to evaluate its association with maternal and pregnancy outcomes. Therefore, the optimal level of 25(OH)D required to prevent adverse outcomes remains to be established in this population. Also, we could not collect sun exposure information from all the participants as it was added to the study protocol after study commencement. Future studies incorporating bone metabolism markers, estimates of calcium excretion rate, and plasma volume expansion, in addition to, investigating pregnancy and birth outcomes would refine the interpretation of PTH–25(OH)D dynamics in pregnancy.

In summary, the vitamin D status of Indian women in early pregnancy can be classified according to the inflection point of 25(OH)D at which PTH is maximally suppressed. Lack of consensus on the current cut-off to identify VDD [50] and absence of antenatal supplementation guidelines in low-resource and high-risk populations, underscore the need to revisit the current recommendations. Thus, the present study serves as an important stepping stone in the field of maternal nutrition, providing critical insights into the relationship of PTH and vitamin D levels of a vulnerable and scarcely studied population—Indian pregnant women. Finally, our findings highlight both modifiable and non-modifiable factors that impact vitamin D status during the first trimester of pregnancy. Future longitudinal and robust trials are warranted to establish causality between vitamin D thresholds and pregnancy-related adverse events to inform clinical and public health interventions.

## 5. Conclusions

Assessing vitamin D status may be challenging due to the influence of various modifiable and non-modifiable factors such as parity, anemia, season, and physical activity. Amidst a lack of national-level data indicating the true burden of VDD, our study demonstrates that vitamin D thresholds defined in association with serum PTH concentration can serve as a reasonably sensitive indicator of VDD. Establishing similar population-specific thresholds can prevent overestimation of VDD prevalence especially in settings with limited resources. Further, identifying this threshold during early gestation offers a critical opportunity to inform future trials and, ultimately, guide antenatal supplementation programs in India.

## Figures and Tables

**Table 1:** General characteristics of study participants.

Variables (Mean ± SD)	N = 396
** Age (years) **	26.3 ± 4.0
** Gestational age **	11.6 ± 2.3
** Education, ** *** n *** ** (%) **	
Up to high school	50 (12.6%)
PUC and diploma	98 (24.7%)
University and above	248 (62.6%)
** Parity, ** *** n *** ** (%) **	
Nulliparous	250 (63.1%)
Multiparous	146 (36.9%)
** Participant employment, ** *** n *** ** (%) **	
Yes	127 (32.1%)
No	269 (67.9%)
** Anthropometry **	
Weight (kg)	57.5 ± 11.0
Height (cm)	155.7 ± 5.4
Body Mass Index (kg/m^2^)	23.7 ± 4.2
** Body Composition **	
Fat percent	29.3 ± 3.2
Fat mass (kg)	17.1 ± 4.7
Fat-free mass (kg)	40.2 ± 6.3
** Blood pressure (mmHg) **	
Systolic	97.9 ± 9.5
Diastolic	68.4 ± 17.2
** Dietary intakes **	
Energy (kcal/d)	1860 ± 471
Protein (g/d)	57.4 ± 16.3
Total fat (g/d)	54.3 ± 17.6
Carbohydrates (g/d)	285.5 ± 75.4
Fortified Vitamin D_2_ (IU/d)	174.9 (87.2)
Calcium intake (mg/d)	894.5 ± 319.4
Iron (mg/d)	14.8 ± 4.9
** Physical Activity Level (PAL) **	1.5 ± 0.2

**Table 2:** Biochemical profile of study participants at recruitment.

Variable (N = 395)	Category	Mean ± SD
**Hemoglobin (g/dL)**	–	11.8 ± 1.3
**Anemia Status, ** ***n* (%)**	Normal	326 (83.0)
	Mild	37 (9.3)
	Moderate	30 (7.6)
	Severe	3 (0.8)
**Parathyroid Hormone (pg/mL)**	–	24.7 ± 12.4
**Total 25(OH)D (ng/mL)**	–	14.6 ± 8.7

**Table 3:** Associations of serum 25(OH)D status at recruitment: Logistic regression analysis (n = 395).

Covariates	Unadjusted Odds Ratio (95% CI)	*p*-Value
** Season **		
Monsoon	Ref	
Post-Monsoon	3.00 (1.62–5.55)	<0.001
Winter	2.43 (1.41–4.19)	0.001
** Parity **		
Multiparous	Ref	
Nulliparous	1.74 (1.14–2.67)	0.011
** Anemia status **		
Normal	Ref	
Moderate/Severe Anemia	2.76 (1.03–7.41)	0.044
** Physical Activity **	0.24 (0.06–0.97)	0.045

## Data Availability

The datasets presented in this article are not readily available because the data are part of an ongoing study. Requests to access the datasets should be directed to the corresponding author.

## Supplementary Materials

The following supporting information can be downloaded at https://www.mdpi.com/article/10.3390/nu17162626/s1, Table S1: Covariates of serum 25(oh)d and parathyroid hormone levels at recruitment: linear regression results (n = 395). Figure S1: Season-wise average total 25(OH)D and parathyroid concentrations. Figure S2: Ratio of PTH/total 25(OH)D versus total 25(OH)D concentration (ng/mL).

## Abbreviations

The following abbreviations are used in this manuscript:

VDD	Vitamin D Deficiency
PTH	Parathyroid Hormone
IEC	Institutional Ethics Committee
CTRI	Clinical Trials Registry of India
HMSC	Health Ministry’s Screening Committee
HIV	Human Immunodeficiency Virus
HBsAg	Hepatitis B Surface Antigen
EDTA	Ethylenediaminetetraacetic Acid
