# Supplementary material for: Relationship Between Total 25-Hydroxyvitamin D and Parathyroid Hormone Concentrations During Early Gestation in Indian Women
Source: Nutrients. 2025 Aug 14;17(16):2626. doi: 10.3390/nu17162626 (PMC12389060; doi:10.3390/nu17162626)
Supplement: Supplementary file 1 [file nutrients-17-02626-s001.zip › Supplementary Table S1.pdf]

Supplementary Table

Supplementary Table 1. Covariates of Serum 25(OH)D and Parathyroid Hormone Levels at Recruitment: Linear Regression Results (n = 395)

Dependent Variable	Independent Variable	Beta	95% CI	p-value
**Parathyroid Hormone**	Age	-0.058	-0.369 to 0.252	0.713
	**Gestational Age**	**-0.792**	**-1.313 to -0.271**	**0.003**
	**Nulliparous**	**-2.951**	**-5.485 to -0.418**	**0.023**
	Education (Graduation & above)	1.889	-1.895 to 5.673	0.328
	Maternal BMI	0.312	0.022 to 0.601	0.035
	**Weight**	**0.107**	**-0.004 to 0.218**	**0.059**
	Fat Mass	0.19	-0.073 to 0.453	0.158
	**Fat Free Mass**	**0.204**	**0.006 to 0.402**	**0.044**
	Physical Activity Level	5.897	-2.297 to 14.090	0.159
	Calcium Intake	0	-0.004 to 0.004	0.983
	Calculated Vitamin D Intake	0.005	-0.009 to 0.019	0.519
	Haemoglobin	0.434	-0.538 to 1.406	0.382
	Summer	-0.602	-4.627 to 3.422	0.769
	Post-Monsoon	0.679	-2.835 to 4.193	0.705
	Winter	2.153	-1.082 to 5.388	0.193
**Total 25-Hydroxyvitamin D**	Age	0.09	-0.125 to 0.305	0.414
	Gestational Age	0.227	-0.139 to 0.593	0.225
	Nulliparous	-1.172	-2.940 to 0.597	0.195
	Education (Graduation and above)	-0.332	-2.972 to 2.307	0.805
	Maternal BMI	-0.169	-0.371 to 0.032	0.101
	Weight	-0.045	-0.122 to 0.033	0.259
	Fat Mass	-0.069	-0.256 to 0.119	0.472
	Fat Free Mass	-0.088	-0.229 to 0.053	0.222
	Physical Activity Level	4.639	-1.058 to 10.336	0.111
	Calcium Intake	0.001	-0.002 to 0.003	0.577
	Calculated Vitamin D Intake	-0.008	-0.018 to 0.001	0.094
	Haemoglobin	0.09	-0.585 to 0.765	0.793
	Summer	-0.139	-2.874 to 2.595	0.920
	**Post-Monsoon**	**-4.529**	**-6.915 to -2.143**	**0.000**
	**Winter**	**-3.624**	**-5.819 to -1.428**	**0.001**
