# Supplementary figures and images for: Relationship Between Total 25-Hydroxyvitamin D and Parathyroid Hormone Concentrations During Early Gestation in Indian Women
Source: Nutrients. 2025 Aug 14;17(16):2626. doi: 10.3390/nu17162626 (PMC12389060; doi:10.3390/nu17162626)

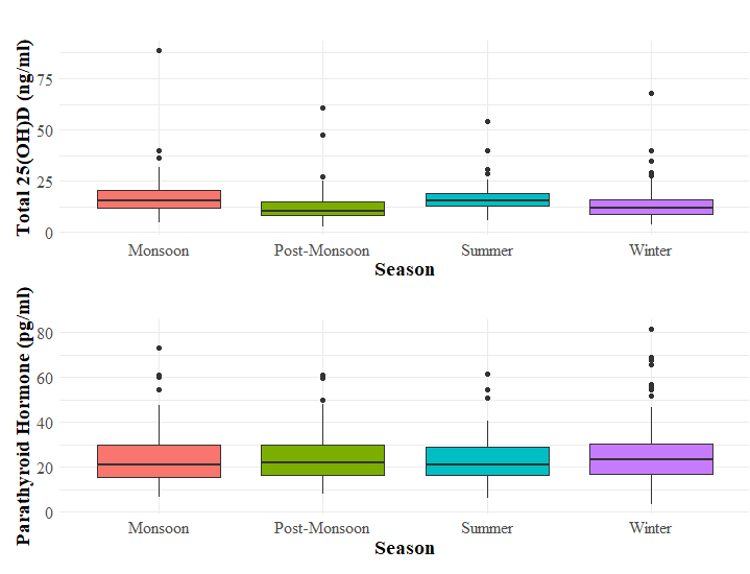

Supplement: Supplementary file 1 [file nutrients-17-02626-s001.zip › Supplementary Figure S1.png]

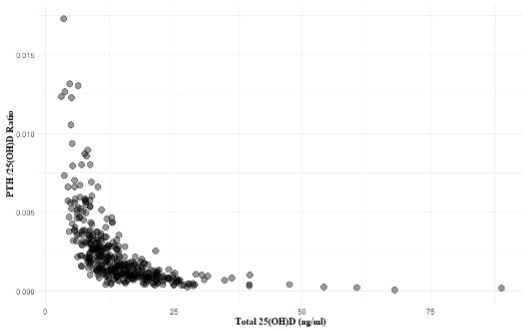

Supplement: Supplementary file 1 [file nutrients-17-02626-s001.zip › Supplementary Figure S2.jpg]
